# Electronic Beam-Scanning Antenna Based on a Reconfigurable Phase-Modulated Metasurface

**DOI:** 10.3390/s22134990

**Published:** 2022-07-01

**Authors:** Zakaria Zouhdi, Badreddine Ratni, Shah Nawaz Burokur

**Affiliations:** 1 LEME, UPL, Université Paris Nanterre, F92410 Ville d’Avray, France; zakaria.zouhdi@naval-group.com (Z.Z.); beratni@parisnanterre.fr (B.R.); 2 Naval Group, Naval Research, 83190 Ollioules, France

**Keywords:** reconfigurable antenna, metasurface, varactor diode, beam-scanning, high gain

## Abstract

Metasurfaces (MSs) have enabled the emergence of new ideas and solutions in the design of antennas and for the control of electromagnetic waves. In this work, we propose to design a directional high-gain reconfigurable planar antenna based on a phase-modulated metasurface. Reconfigurability is achieved by integrating varactor diodes into the elementary meta-atoms composing the metasurface. As a proof of concept, a metasurface prototype that operates around 5 GHz is designed and fabricated to be tested in an antenna configuration. The metasurface is flexibly controlled by different bias voltages applied to the varactor diodes, thus allowing the user to control its phase characteristics. By assigning judiciously calculated phase profiles to the metasurface illuminated by a feeding primary source, different scenarios of far-field patterns can be considered. Different phase profiles are tested, allowing us to, firstly, achieve a highly directive boresight radiation and, secondly, to steer the main radiated beam towards an off-normal direction. The whole design process is verified by numerical simulations and is validated experimentally by far-field antenna measurements. The proposed metasurface enables the design of directive flat antennas with beam-scanning characteristics without complex feeding systems and power-consuming phase shifters, and thus provides potential interests for next generation antenna hardware.

## 1. Introduction

Connectivity technologies continue to evolve year after year to meet growing global demand for increasing data rates and number of connected devices. Indeed, today, not only are humans connected to the global network but objects, sensors, industrial machines, drones and even satellites are all connected to the same network, which is pushing further the development of new technologies that allow all these needs to be met. As such, the development of a new generation of reconfigurable antennas enabling real-time monitoring of users is necessary.

The emergence of artificially structured materials, called metamaterials, has revolutionized the domain of electromagnetism. These engineered structures were first used in their three-dimensional (3D) configuration and demonstrated several phenomena and concepts [1,2,3,4,5]. Over the years, the community has become more and more interested in the development of two-dimensional (2D) versions of metamaterials, commonly known as metasurfaces. Such planar versions reduce the volume and complexity of realization and losses in comparison to the bulky 3D counterparts [6]. Thanks to these structures, new properties, such as anomalous reflection and refraction, have been proposed and demonstrated [7,8,9]. Metasurfaces also facilitated the generation of complex wavefronts, such as Airy beams [10,11], vortex beams carrying orbital angular momentum (OAM) [12,13], Bessel beams [14,15,16] and image holograms [17,18,19,20,21]. In the field of antennas, metasurfaces have aided in developing different topologies of structures. For instance, the dimensions of antennas can be drastically reduced, particularly when they are used as partially reflective surfaces (PRS) in reflex-type Fabry–Perot (FP) cavity antennas to achieve high directivity from a single radiating element [22,23]. Flat lenses [24,25], leaky-wave antennas [26] and polarizers [27,28] have also been designed based on the use of metasurfaces. In order to reach real-time control of the electromagnetic properties, as required in reconfigurable antennas, lumped electronic components are loaded in meta-atoms composing the metasurfaces [29]. Several types of components have been considered for reconfigurability mechanism, such as liquid crystals [30,31], PIN diodes [32,33], varactor diodes [15,34], MEMS [35] and graphene [36]. The electronic elements are used to tune the resonant properties of the meta-atom, thereby helping to dynamically tailor electromagnetic wavefronts. In the field of antennas, one of the applications that necessitates reconfigurable metasurfaces is beam scanning, in which the main beam is electronically steered through tailoring the phase profile implemented in the metasurface [37,38,39].

In this work, we propose a dynamic metasurface loaded with varactor diodes for an operation around 5 GHz frequency. The metasurface controlled by a direct current (DC) bias voltage is exploited as a reconfigurable planar reflector with a parabolic phase profile in order to design a highly directive antenna. Such antenna allows reconfigurability in frequency, thus making it possible to cover a non-negligible operating frequency band. It further allows control of the direction of the radiated main beam in order to achieve beam steering. A prototype of the reconfigurable metasurface is fabricated and experimentally tested in a reflect-array antenna configuration in a microwave anechoic chamber in order to validate its performances and functionality. Such a dynamic metasurface-based antenna platform is an interesting alternative to transmit highly directive but also continuously steerable beams at large angles, thus showing great potential in wireless communications and smart antennas where real-time control is desired.

## 2. Design Principle

**Figure 1:**
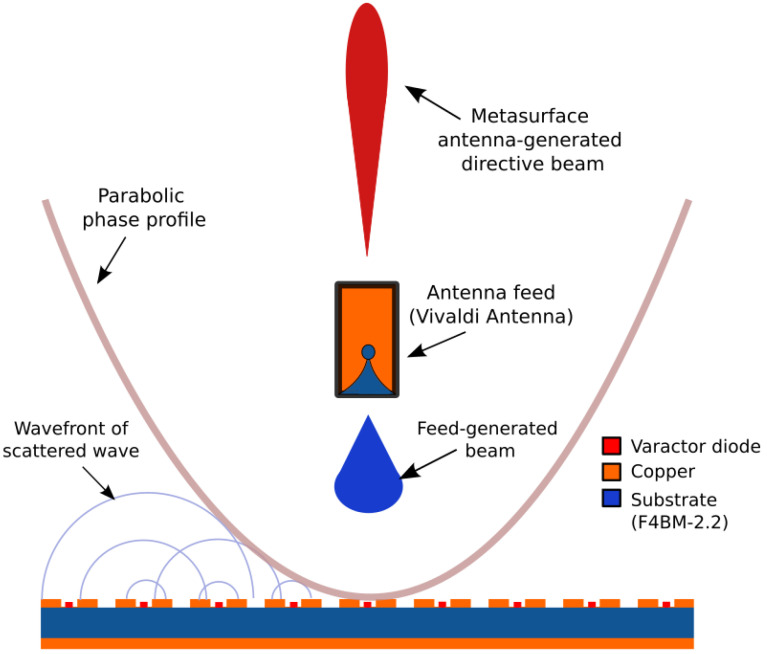
Schematic illustration of the operating principle of the flat meta-reflector antenna. The meta-reflector presents a parabolic phase profile and is illuminated by a radiating element used as primary feed. The resulting far-field radiation pattern of such an antenna configuration is a directive beam.

The main goal of this work is to design a flat metasurface reflector (or meta-reflector) antenna. The meta-reflector is intended to have a parabolic phase profile and to be illuminated by a radiating element used as primary feed, as schematically illustrated in Figure 1. In order to emulate the response of a parabolic reflector, the reflection caused by the metasurface must be identical to that of a parabola. For this, we start by calculating the necessary phase profile that allows to mimic a parabolic reflector. The parabolic phase profile *φ*(*x*, *y*) for a given operating wavelength *λ* and focal distance *F* is given as [40]:

(1) $$\varphi \left(x,y\right)=\frac{2\pi }{\lambda }\left(\frac{{\left(x-x_{0}\right)}^{2}+{\left(y-y_{0}\right)}^{2}}{4F}\right)+\varphi_{0}$$

where (*x*_0_, *y*_0_) is the focal point position and *φ*_0_ is the reflection phase at (*x* = 0, *y* = 0).

Equation (1), therefore, describes a parabolic phase profile, as illustrated in Figure 1. Considering the reconfigurability mechanism of the metasurface, the phase profile can be tuned according to the wavelength, focal distance and focal point position. Consequently, the meta-reflector antenna would cover a non-negligible frequency band of operation, as well as steer the reflected beam. For smart systems, it is preferable that the antenna should be as compact as possible with a small focal distance. In the case of parabolic antennas, in order to maximize the gain, it is further desirable to maximize the illumination on the reflector by the primary feed source.

## 3. Design of the Metasurface Reflector

**Figure 2:**
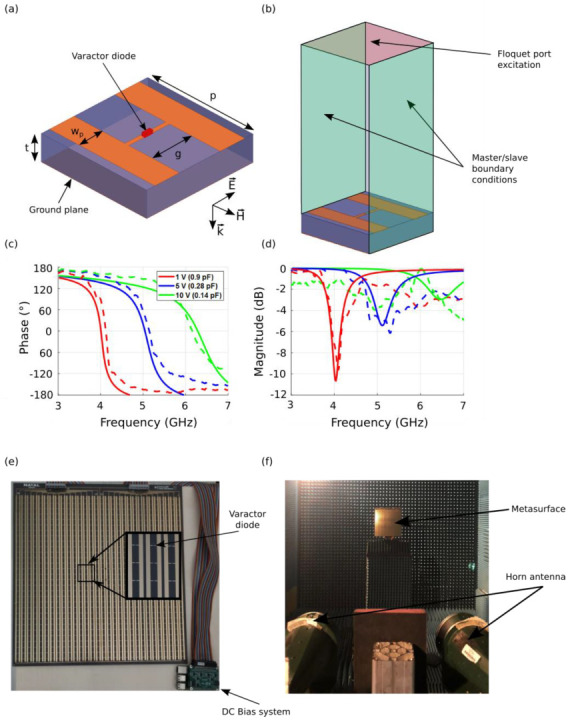
Design of the reconfigurable metasurface reflector. (**a**) Schematic view of the elementary meta-atom incorporating a voltage-controlled varactor diode and (**b**) schematic view of the simulation environment. The substrate used has a relative permittivity *ε*_r_ = 2.2 and a thickness *t* = 3 mm. The geometrical dimensions are: *p* = 13 mm, *w*_p_ = 3 mm and *g* = 5 mm. Simulated and measured reflection responses for different applied stimuli signals (capacitance values in simulations and bias voltages in experiments): (**c**) phase and (**d**) magnitude. Photograph of the fabricated metasurface (**e**) and the experimental characterization setup (**f**).

For the design of the metasurface that will serve as a reconfigurable reflector, we start by designing the elementary meta-atom. The main requirement is that the meta-atom presents an inductive-capacitive (*LC*) resonance that allows achieving a quasi-360° phase shift together with a high reflectivity. The considered unit cell structure has a periodicity *p* = 13 mm and is composed of two metal layers printed on the faces of a F4BM dielectric substrate with a relative permittivity *ε*_r_ = 2.2 and thickness *t* = 3 mm. The top layer is composed of two parallel copper strips of width *w*_p_ = 3 mm, which are separated by a gap *g* = 5 mm, while the bottom layer is composed of a continuous ground plane, as shown in the schematic view presented in Figure 2a. The separation gap *g* between the parallel strips of the top layer allows for a capacitive response when the electric field is oriented perpendicular to the strips. Additionally, the continuous ground plane contributes to produce an inductive response and to fully reflect the incident electromagnetic wave. The combination of the capacitive and inductive layers therefore achieves the desired *LC*-resonant circuit.

In order to electronically control the response of such a *LC*-resonant meta-atom, a varactor diode that serves to modify the capacitance is loaded in the elementary meta-atom. A DC bias voltage is applied to the varactor diode through the two parallel copper strips of the capacitive layer. A MAVR-000120-1411 varactor diode model [41] is considered due to its low losses and high tuning factor. In order to simulate the behavior of the elementary controllable meta-atom, the varactor diode is modelled as a *RLC* series circuit, where *R* = 3.5 Ω represents the ohmic losses, *L* = 0.9 nH is the inductance due to the packaging and *C* is the overall capacitance of the structure. The meta-atom structure is simulated using the finite element method (FEM) of Maxwell’s equations from the commercially available high-frequency structure simulator (HFSS) code by Ansys [42]. Periodic boundary conditions and Floquet ports are utilized in the simulation setup in order to consider an infinite array of meta-atoms, as presented in Figure 2b. The influence of the variable capacitance on the meta-atom is illustrated in Figure 2c, where the frequency response for different capacitance values, are presented. The simulation results show that the varactor diode allows a shift of the resonance frequency from 4.2 GHz to 5.5 GHz, when the capacitance varies from 0.6 pF to 0.2 pF. The *LC* resonance feature can also be clearly observed with a reflection phase varying from +180° to −180° and passing through 0° at the resonance frequency.

A prototype of the reconfigurable metasurface is realized using a conventional printed circuit board (PCB) manufacturing process. A photograph of the fabricated prototype is shown in Figure 2e, together with the experimental characterization setup (Figure 2f). The metasurface is composed of 30 columns, each containing 30 resonant unit cells (30 × 30 cells), and has lateral dimensions 390 mm × 390 mm (6.5*λ*_0_ × 6.5*λ*_0_ at 5 GHz). In this voltage bias configuration, where a similar voltage will be applied to all the meta-atoms in a column, only a one-dimensional phase profile can be tailored. As such, we are restricted to cylindrical parabolic phase profile instead of a full parabolic phase profile. However, such cylindrical parabolic phase distribution is considered enough to meet the requirements of a proof-of-concept prototype.

The metasurface is characterized in a microwave anechoic chamber, where two broadband FLANN DP240 horn antennas [43] operating in the 2–18 GHz frequency band are connected to the vector network analyzer (VNA) and positioned side by side in front of the metasurface in order to measure its reflection coefficient, as shown in Figure 2f. The reflection coefficient is obtained by measuring the amplitude of the transmitted wave between the two horn antennas after being reflected by the metasurface. The reflection measurements performed on the metasurface are referenced with respect to that on a metal plate with similar lateral dimensions. The simulation and measurement results are presented in Figure 2c, where the correspondence between bias voltage and capacitance value is shown. It is important to note that in such a characterization procedure, all bias voltages are set at the same value throughout the metasurface. As illustrated for a different set of values, 1 V (*C* = 0.6 pF), 4 V (*C* = 0.28 pF) and 8 V (*C* = 0.2 pF), a good qualitative agreement is obtained, thus validating the concept of the reconfigurability mechanism implemented in the metasurface. A decrease in the capacitance value of the varactor diode induced by an increase in bias voltage leads to a resonance shift toward higher frequencies, which enables a shift in the phase response of the metasurface. This phase shift is very important since it will allow the tailoring of the phase distribution required to mimic the profile of a parabola. A high phase shift Δ*φ* (above 280°) is achieved within the 4.4–5 GHz due to the intrinsic design of the meta-atom. With such phase-tuning capability, it is therefore possible to generate a cylindrical parabolic phase profile from the metasurface.

## 4. Primary Feed Design

**Figure 3:**
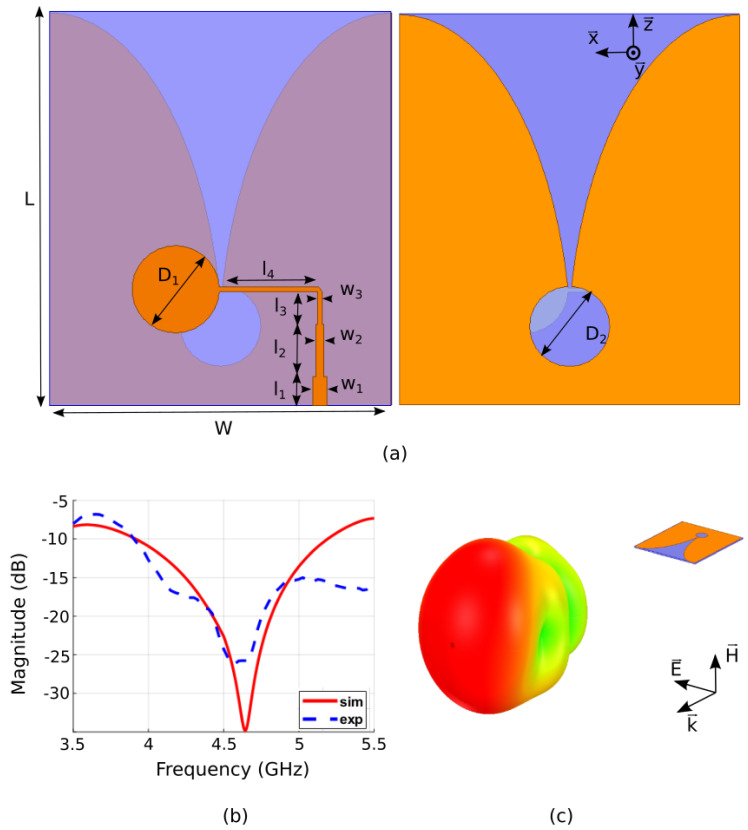
Feeding source used to illuminate the meta-reflector. (**a**) Schematic view of the Vivaldi antenna structure (top and bottom layers). The geometrical dimensions are: *L* = 83 mm, *W* = 72 mm, *l*_1_ = 6 mm, *l*_2_ = 11 mm, *l*_3_ = 7 mm, *l*_4_ = 21 mm, *w*_1_ = 3 mm, *w*_2_ = 1.8 mm, *w*_3_ = 0.9 mm, *D*_1_ = 17 mm and *D*_2_ = 9.2 mm. (**b**) Simulated (blue trace) and measured (red trace) reflection responses (*S*_11_ coefficients) of the proposed primary source. (**c**) Schematic view of the Vivaldi antenna along with its 3D radiation pattern.

A primary feed is required to illuminate the metasurface reflector. This radiating element positioned at a certain distance in front of the metasurface will launch electromagnetic waves, which will be reflected by the meta-reflector. It is therefore necessary that the feeding source does not mask the reflected waves. Here, we therefore propose to use a Vivaldi radiating element as primary source. The choice of such a type of source is made for two reasons; the first one is due to its end-fire radiation characteristics, i.e., the maximum radiation lies in the plane of the radiating element, which considerably reduces the masking effect mentioned above, while the second reason is that such antennas present broadband features and, therefore, allow covering a wide frequency band of operation. A schematic view of the proposed Vivaldi radiating element printed on a F4BM dielectric substrate with relative permittivity *ε*_r_ = 2.2 and thickness *h* = 1 mm is shown in Figure 3a. The antenna is optimized in numerical simulations for an operation around 5 GHz. The geometry of the source is based on the coplanar Vivaldi antennas with the Vivaldi design on the upper layer of the substrate composed of an exponentially tapered slot [44,45]. The feed of the Vivaldi antenna is composed of a microstrip line transition to a radial slot of diameter *D*_1_ with a circular stub of diameter *D*_2._ The optimized geometrical dimensions of the radiating element on the top layer are *D*_1_ = 17 mm, *L* = 83 mm and *W* = 72 mm for the top layer. The bottom layer is composed of the transmission-line excitation system, which is composed of a 50 Ω line with length *l*_1_ = 6 mm and width *w*_1_ = 3 mm. In order to match the input impedance of the excitation line, a quarter-wavelength transformer of length *l*_2_ = 11 mm and width *w*_2_ = 1.8 mm is added. The last part of the line, with *l*_3_ = 7 mm, *l*_4_ = 21 mm and *w*_3_ = 0.9 mm, is connected to an offset feed of diameter *D*_2_ = 9.2 mm.

It should also be noted that in the reflector antenna scenario, the Vivaldi radiating element will be in close proximity to the metasurface. Therefore, it has to be designed and optimized by taking into account the coupling with the metasurface. The antenna is fabricated using PCB technique and measured in an anechoic chamber. The experimental reflection coefficient is compared to the simulated one in Figure 3b. A good agreement is observed between the two results and a good impedance matching (*S*_11_ < −10 dB) is observed over a wide frequency, ranging from 3.9 GHz to 5.2 GHz in simulation, while the measured result shows a good impedance matching from 3.9 GHz to above 5.5 GHz. This result is excellent in our case since the desired frequency band of operation of our meta-reflector antenna is from 4.4 GHz to 5 GHz. It will be shown in the next section that when placed in front of the metasurface, the reflection coefficient of the reflector antenna system remains quasi-similar to the feeding Vivaldi element alone. The simulated 3D radiation pattern of the Vivaldi radiating element is shown in Figure 3c, where a unidirectional beam can be observed.

**Figure 4:**
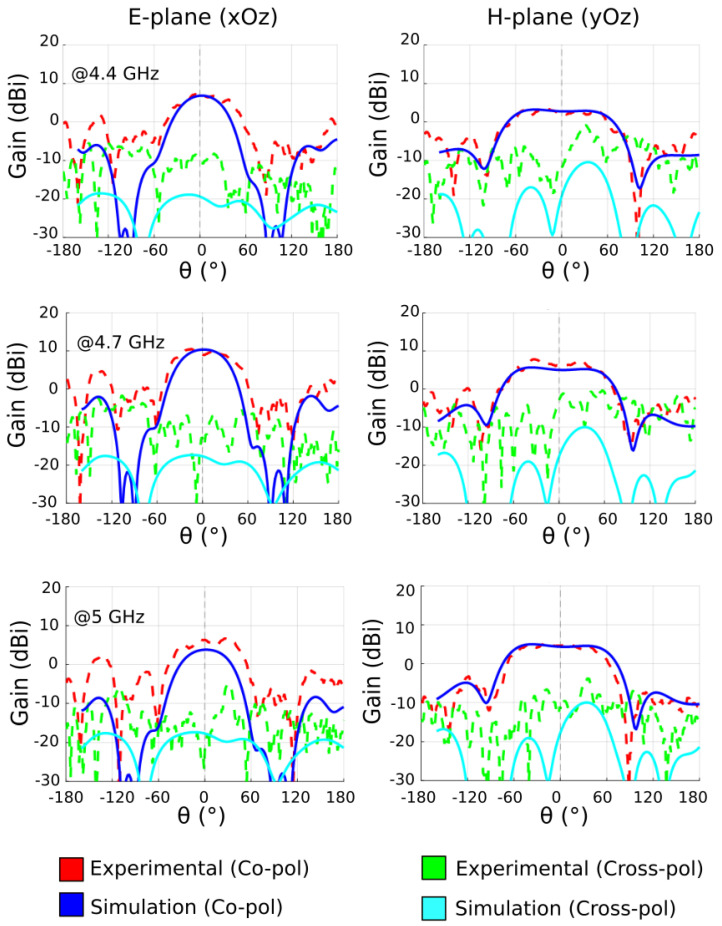
Simulated and measured *E*-plane and *H*-plane radiation patterns of the Vivaldi radiating element at 4.4 GHz, 4.7 GHz and 5 GHz.

In Figure 4, the 2D far-field radiation patterns are shown at 4.4, 4.7 and 5 GHz, where the blue solid line and red dotted line are the simulated and measured co-polarized gain (*E*-plane patterns), respectively. The simulation shows good agreement with the experiment as the maximum gain is measured to be 7 dBi at 4.4 GHz, 9.8 dBi at 4.7 GHz and 5.7 dBi at 5 GHz. The half-power beamwidth is measured to be approximately 60° at the three tested frequencies. The cross-polarized gains (*H*-plane patterns), represented by the cyan and green traces, show relatively low level.

The difference in side-lobes between simulated and experimental results in the *E*-plane is caused by the coaxial cable feeding the Vivaldi antenna. The end-fire configuration of such a Vivaldi antenna seems to be the reason for such a phenomenon, which then has some influence on the side lobes of the metasurface antenna.

## 5. Design of the Flat Meta-Reflector Antenna

**Figure 5:**
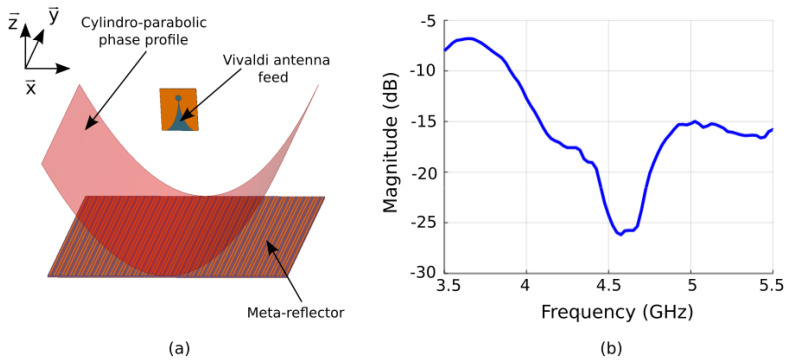
(**a**) Schematic view the reconfigurable flat meta-reflector antenna. (**b**) Measured reflection coefficient.

Once the primary source and the metasurface have been designed and experimentally characterized separately, the meta-reflector antenna is assembled, as illustrated in Figure 5a. For an experimental proof-of-concept prototype of the reconfigurable meta-reflector, we limit the parabolic phase profile to only a one-dimensional (1D) plane, as restricted by the design of the metasurface, which can be controlled in a single plane. Therefore, the phase profile is applied only along the *x*-axis. The full parabolic phase distribution of Equation (1) is then simplified to a cylindrical parabolic one as:

(2) $$\varphi \left(x\right)=\frac{2\pi }{\lambda }\frac{{\left(x-x_{0}\right)}^{2}}{4F}+\varphi_{0}$$

In our case, as the metasurface has lateral dimensions of 390 mm × 390 mm, and with the HPBW (half-power beamwidth) of the antenna being 60°, a focal distance should be considered such that the metasurface is correctly illuminated by the main beam. The antenna system is simulated using Ansys HFSS and after several optimization simulations, the focal distance *F* is set to 120 mm. Imposing 120 mm as a focal distance is mainly motivated by a trade-off between two opposing factors. A higher *F*/*D* ratio (*D* being the metasurface dimension) would lead to an improvement in gain, whereas a lower one would reduce the antenna’s profile but with a reduced gain. It is important to note that while a large focal distance would yield an increase in aperture efficiency, the structure is being designed for a low-profile perspective in the present study.

The reflection coefficient of the meta-reflector antenna system is measured and compared to the simulation results, as presented in Figure 5b. A good agreement between measurements and simulations is obtained and a good impedance matching is observed in our frequency band of interest between 4.4 GHz and 5 GHz, where the reflection coefficient shows amplitude values lower than −10 dB.

**Figure 6:**
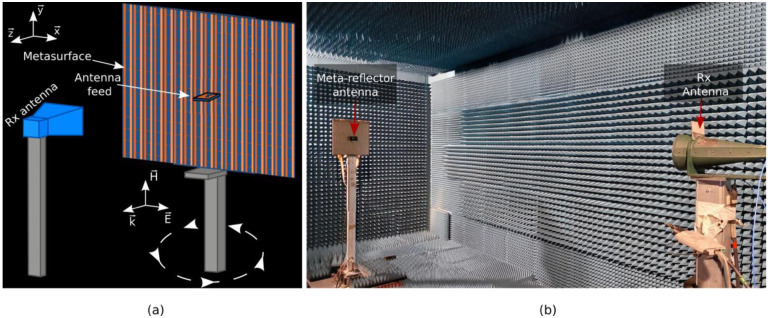
Antenna radiation patterns measurement. (**a**) Schematic illustration of the experimental measurement setup. The meta-reflector is illuminated by the Vivaldi radiating element and a receiving horn antenna allows measurement of the antenna radiation patterns. (**b**) Photograph of the far-field measurement setup in an anechoic chamber.

**Figure 7:**
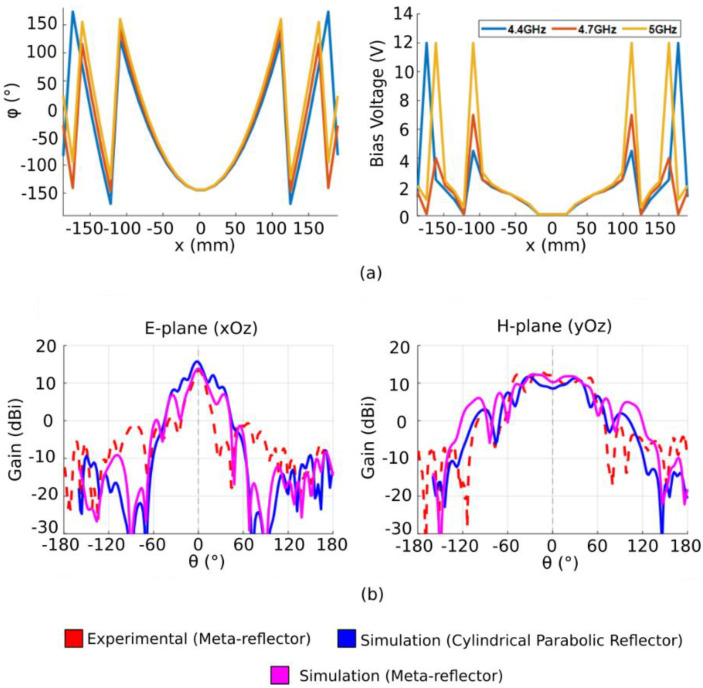
(**a**) Calculated phase profiles and applied bias voltages at 4.4 GHz, 4.7 GHz and 5 GHz. (**b**) Radiation patterns of the fully simulated gradient metasurface structure at 4.7 GHz, the metallic cylindrical reflector antenna simulation and the experimental measurements of the gradient meta-reflector.

In order to validate the proposed flat meta-reflector antenna, measurements of the radiation patterns are performed in an anechoic chamber. A schematic view of the measurement setup is presented in Figure 6a and a photograph of the antenna in the test environment is shown in Figure 6b. The antenna under test is placed on a turntable and is connected to one port (port 1) of the VNA, while the (2–18 GHz) FLANN broadband horn antenna connected to the other port (port 2) of the VNA is used as a receiving antenna at a distance of 6 m. The antenna system under test on the turntable is rotated between from −180° to +180° in order to measure the transmitted power between the two antennas and therefore the antenna far-field radiation patterns. Several antenna configurations are tested. The cylindrical parabolic phase profiles allowing a boresight radiation to be obtained at 4.4 GHz, 4.7 GHz and 5 GHz are calculated from Equation (2) by fixing *φ*_0_ = 0° and are plotted in Figure 7a. These phase profiles are applied to the metasurface by judiciously applying the correct bias voltage corresponding to the required capacitance value to each column of meta-atoms. For large antennas, typical full-wave solvers require significantly large simulation time and memory constraints. As a way to deal with this limitation, the metasurface is approximated by its ideal case, i.e., a metallic cylindrical parabolic reflector antenna. In order to verify the accurateness of this approximation, the phase-modulated metasurface is fully simulated at 4.7 GHz and compared to the subsequent metallic cylindrical parabolic case, as well as the experimental measurements. The results, as shown in Figure 7b, display a clear correlation between the cylindrical parabolic reflector antenna, the fully simulated gradient metasurface and experimental results, thus validating the approximation, which will be used henceforward.

**Figure 8:**
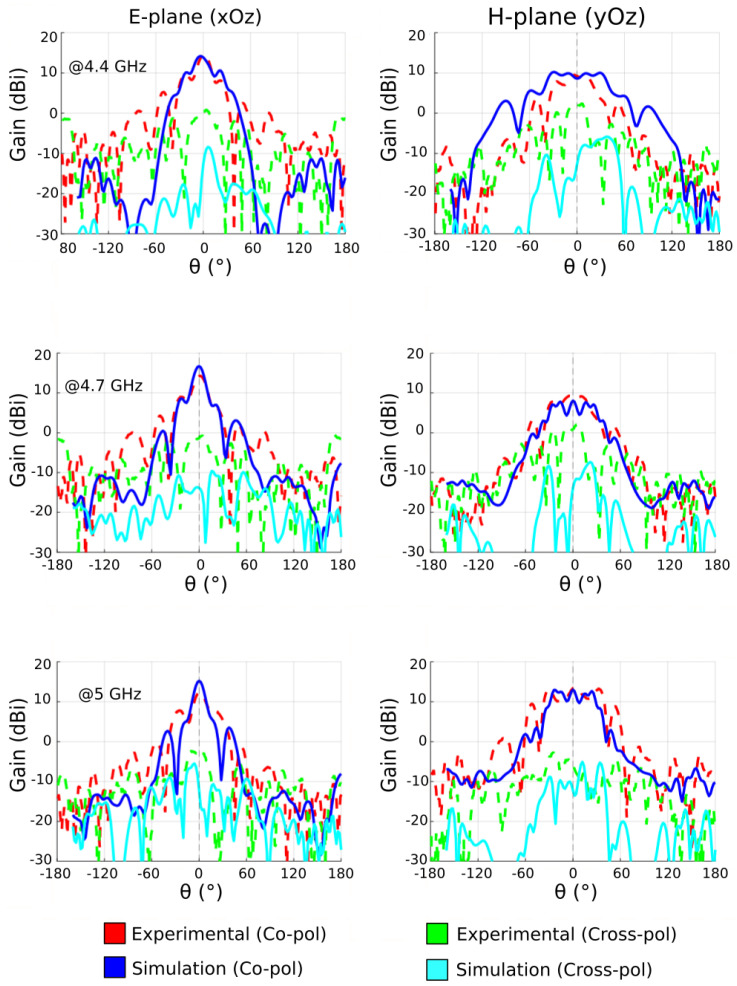
Simulated and measured *E*-plane radiation patterns of the meta-reflector antenna at 4.4 GHz, 4.7 GHz and 5 GHz.

The measured radiation patterns are presented in Figure 8 and show a highly directive beam in the *E*-plane (*xOz* plane), where the phase profile is applied. The maximum gain reaches 16 dBi at the central frequency of 4.7 GHz. The half-power beamwidth of the antenna is found to be around 32°. In the *H*-plane (*yOz* plane), the patterns are similar to those of the Vivaldi feeding source since no parabolic phase profile is applied in this plane. A difference of minimum 12 dB can be observed between the co- and cross-polarized gains, indicating a sufficient decoupling. The presented results allow the validation of the proposed flat parabolic reflector antenna concept by comparing the experimental measurements with a simulated metallic cylindrical parabolic reflector antenna.

**Figure 9:**
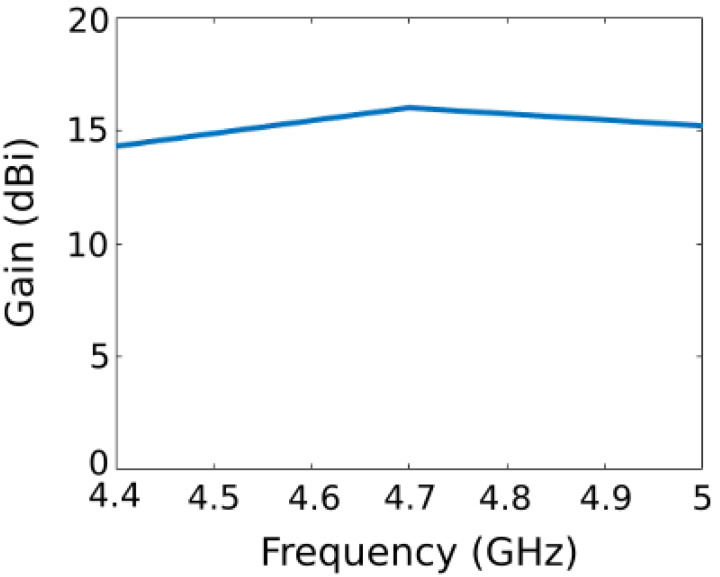
Experimental gain at boresight versus frequency of the meta-reflector antenna.

Moreover, the gain at boresight is measured and is presented in Figure 9. At 4.7 GHz, the gain reaches 16 dBi. The aperture efficiency *η* can be calculated as [44]:

(3) $$\eta =\frac{A_{e}}{A}=\frac{G\lambda^{2}}{4\pi A}$$

with *A_e_* being the effective aperture, *A* the physical aperture (390 mm × 390 mm) and *G* the experimentally measured gain at wavelength *λ*. The calculated aperture efficiency at 4.4 GHz, 4.7 GHz and 5 GHz are, respectively, 6%, 7% and 8%. Comparatively, the aperture efficiency of a parabolic reflector is typically around 50% to 70%. The reason for this big difference is explained by different factors. Firstly, the proposed metasurface antenna presents gain values ranging from 14 dBi to 16 dBi. These low gains are mostly due to the fact that the meta-reflector has been engineered to produce a cylindrical parabolic phase profile (i.e., a parabolic phase profile in only one plane) instead of a full parabolic phase profile, where the gain would be above 20 dBi. Another important issue is that we use electronic components, namely varactor diodes, in the meta-reflector. These varactor diodes have a certain parasitic resistance that will partly absorb electromagnetic waves. Thus, the gain is lower with the reconfigurable meta-reflector. The second factor is justified by our choice of focal distance *F* = 120 mm so as to achieve a low-profile antenna. Consequently, the best trade-off that allows the gain to be maximized while reducing the profile of the antenna as much as possible is to place the illuminating source at a focal distance of 120 mm from the reflector.

The illumination efficiency *η_i_* and spillover efficiency *η_s_* have also been calculated using [46]:

(4) $$\eta_{i}=\frac{{\left(\frac{1+\cos^{q+1}\theta }{q+1}+\frac{1+\cos^{q}\theta }{q}\right)}^{2}}{2\tan^{2}\theta \left(\frac{1-\cos^{2q+1}\theta }{2q+1}\right)}$$

(5) $$\eta_{s}=1-\cos^{2q+1}\theta$$

where *q* is the exponent of a ${\cos\;}^{q}\theta$ (with *q* = 3) radiation pattern that approximates the experimentally measured pattern of the Vivaldi source and *θ* is half of the subtend angle from the feed to the reflectarray aperture for a 120 mm focal distance (*θ* = 57° in our case).

At 4.7 GHz, it is found that *η_i_* = 45% and *η_s_* = 98%. This is consistent with the fact that the focal distance is at a sub-optimal configuration and is, therefore, unable to fully illuminate the structure (low illumination efficiency). Meanwhile such a small focal distance leads to a high spillover efficiency.

**Figure 10:**
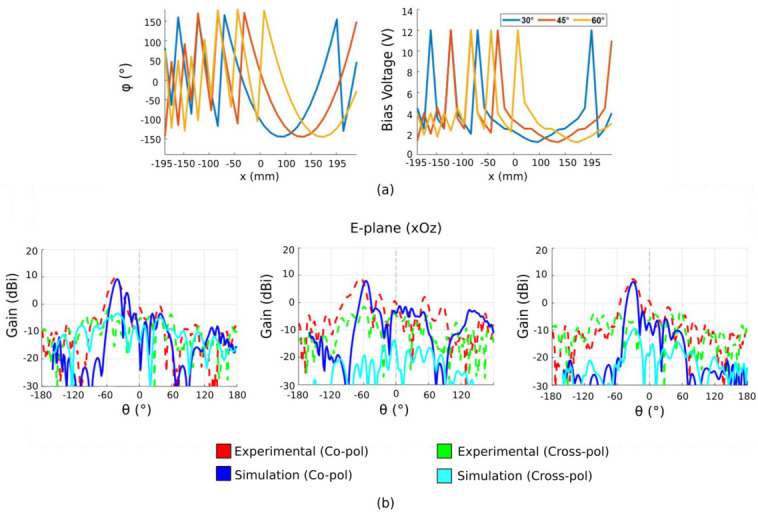
(**a**) Calculated phase profiles and applied bias voltage allowing beam steering at 30°, 45°, 60°. (**b**) Measured radiation patterns along the *E*-plane showing beam steering at 30°, 45°, 60°.

The possibility of controlling the direction of the main radiated beam is also proposed. In the conventional case, it is possible to perform beam steering by physically shifting the feeding source away from the focal point or by turning the parabolic reflector toward the desired direction. However, given that the metasurface allows an electronic control of the phase profile, we are able to introduce an offset *x*_0_ in order to virtually move the parabola with respect to the source and, therefore, achieve beam steering. In order to validate the beam-steering capabilities, the phase profile is shifted progressively. Three different beam-steering angles (30°, 45° and 60°) are tested at 4.7 GHz. The corresponding phase and bias voltage profiles that need to be implemented on the metasurface are shown in Figure 10a. Simulation and experimental results presented in Figure 10b show high beam-steering capabilities up to 60° with side-lobe levels (SLL) lower than 10 dB. The high beam-steering range of the proposed antenna is an improvement over the capabilities of previous gradient metasurfaces presented in literature, where beam steering was achieved up to 50° [8,37]. The parabolic phase profile also enables a significant increase in gain compared to classic beam-steering gradient metasurfaces. Though the radiated beam is steered, it can be clearly observed that a high maximum gain is maintained. However, due to the 1-D property of the phase profile, the half-power beamwidth remains large in the *H*-plane.

**Figure 11:**
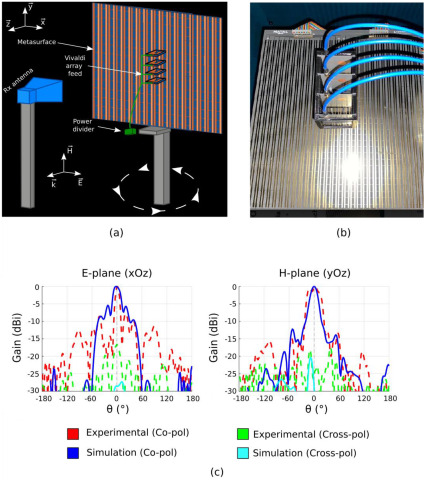
(**a**) Schematic illustration of the experimental measurement setup. (**b**) Photograph of the metasurface with its Vivaldi array feed. (**c**) Simulated and measured far-field radiation patterns at 4.7 GHz in the *E*- and *H*-planes for the meta-reflector fed by the Vivaldi antenna array.

In order to improve the half-power beam width in the *H*-plane and acquire higher gain suitable for 5G applications, a 1 × 4 Vivaldi array of identical antenna elements is proposed to be aligned in the *H*-plane of the antenna platform, as shown in Figure 11a,b.

Experimental results presented in Figure 11c show a high directivity in both *E*- and *H*-planes in the case of boresight radiation configuration. The measured maximum half-power beam width is around 20° in both planes, showing a 65% decrease of the HPBW in the *H*-plane as well as a 38% decrease in the *E*-plane. Consequently, this solution allows the design of a highly directive antenna where only a single beam-scanning plane is desired. Furthermore, the antenna can possibly show beam-steering capabilities in both planes by using the reconfigurable meta-reflector in the *E*-plane and phase shifters in the *H*-plane or designing a metasurface where control of the phase can be achieved in the two planes.

## 6. Conclusions

In summary, a reconfigurable meta-reflector including varactor diodes is proposed. The use of varactor diodes allows continuous control of the phase response of the meta-reflector. The proposed reflector structure is associated with a Vivaldi antenna used as a primary source in order to realize a highly directive reconfigurable antenna. The primary source is designed specifically to take into account the metasurface/feed coupling. A prototype of the meta-reflector antenna has been fabricated and characterized. Far-field measurements have been performed in an anechoic chamber, where several different desired configurations were tested with judiciously calculated phase profiles. Firstly, boresight radiation at different frequencies (4.4 GHz, 4.7 GHz and 5 GHz) were tested, and the experimental results show a highly directive beam with a gain of around 16 dBi. Then, beam-steering functionality was also verified in one radiation plane through the introduction of a lateral shift in the parabolic phase profile. Different phase profiles for different beam-steering capabilities were tested and the results show that remarkably high beam steering can be achieved, with up to 60° in steering while maintaining side-lobe levels under 10 dB. The obtained results for these different tested configurations allowed the validation of the proposed concept. Finally, due to the use of a Vivaldi radiating element as feed to illuminate the reflector, the radiated beam in the *H*-plane shows a large half-power beamwidth. As such, a solution of using a 1 × 4 Vivaldi array of identical antenna elements to reach high directivity also in *H*-plane is proposed.

The resulting performances, particularly the directive nature of the antenna’s radiated beam, pave the way for several applications in the field of 5G, satellite and naval communications. The high beam-steering capabilities allow a hemispheric coverage using a reduced number of antennas. Furthermore, owing to its relatively low profile and simple design, the proposed antenna is a good candidate for integration on planar surfaces, such as walls and ship topside hulls.

Future works will be dedicated to electronic beam-scanning in both *E*- and *H*-planes. Two different solutions can be considered. The first one consists of using a phase shifter on the 1 × 4 array of feeding elements. The second solution consists of developing a meta-reflector platform where the meta-atoms can be controlled individually in such a way that a full parabolic phase profile can be achieved, as well as beam-scanning in both radiation planes.

## Data Availability

Not applicable.
